# Evaluation of the Effect of Titatnium Dioxide Nanoparticles/Gelatin Composite on Infected Skin Wound Healing; An Animal Model Study

**DOI:** 10.29252/beat-070405

**Published:** 2019-10

**Authors:** Amin Nikpasand, Mohammad Reza Parvizi

**Affiliations:** 1 *Department of Physiology, Faculty of Medicine, AJA University of Medical Sciences, Tehran, Iran*

**Keywords:** Wound, Titanium dioxide, Gelatin, Nanoparticle, Rat

## Abstract

**Objective:**

To evaluate effects of titanium dioxide /gelatin nanocomposite on wound healing in mice as a model study.

**Methods:**

Fifty male rats were randomized into five groups of ten animals each. In group I, 0.1 mL sterile saline 0.9% solution was added to the wounds with no infection. In group II, the wounds were infected with MRSA and only treated with 0.1 mL the sterile saline 0.9% solution. In group III, infected wounds were treated with gelatin. In group IV, animals with infected wounds were treated with 0.1 mL titanium dioxide nanoparticles. In group V, animals with infected wounds were treated with titanium dioxide /gelatin nanocomposite. Wound size was measured on 2, 6, 10, 14, 18 and 20 days after surgery.

**Results:**

Reduction in wound area indicated that there was significant difference between group IV and other groups (*p*<0.05). Quantitative histological and morphometric studies and mean rank of the qualitative studies demonstrated that there was significant difference between group IV and other groups (*P*<0.05).

**Conclusion:**

Titatnium dioxide nanoparticles/gelatin composite offered potential advantages in wound healing acceleration and fibroblast proliferation on early days of healing phases. Acceleration in wound repair could be associated with earlier wound contraction and stability of damaged area by rearrangement of granulation tissue and collagen fibers.

## Introduction

Various medications including chemical and herbal drugs have been utilized with the aim of wound healing acceleration [1,2]. Application of nanotechnology has gained progressive interest for regeneration of injured wound tissue. Collagen/gold nanoparticle nanocomposite and nanoparticles like zinc, zinc oxide, silver, titanium dioxide and zeolite can act as efficient drug delivery vehicle for controlled and targeted release or as covering scaffold in combination with synthetic and natural compounds including chitin, chitosan, collagen, keratin, polyethylene and gelatin [3-5]. Gelatin, natural polymer of bone and skin collagen, create great potential advancement as nanomaterial scaffold because of bioavailability, nontoxicity, biodegradability and biocompability properties. Moreover, gelatin bears strong hydrophilic activity and prevent from fluid loss in the wound area, ultimately leading to preserve wound moisture and create positive effect on regeneration of injured tissue [6].

Titanium dioxide, one of mineral oxides, bears particular and unique characteristics such as electrical and photocatalytic effect and have numerous applications [7]. The most important application areas are purification, disinfection, production of particular ceramics, cosmetics, photo catalyst, destroying tumor cells and making protective covering against UV [8]. Titanium dioxide is an ideal photo catalyst through UV absorption feature. Considering the UV absorption effect as well as photocatalytic properties of nano titanium dioxide, the nanoscale material creates antibacterial coat on the covering surfaces and prevent from rat’s transition [9]. The promising effects of titanium dioxide in wound healing process through antimicrobial and cell growth stimulation features have been reported by others [6,10,11]. The priorities of wound healing focus on acceleration of the healing process as well as preventing from the wound infections. Thus, nano titanium dioxide with having both properties is expected to create favorable consequences on wound healing and optimizing functional recovery [10]. 

Methicillin-resistant *Staphylococcus aureus* (MRSA) is the most widespread bacterial pathogen causing various infections ranging from skin and soft tissue infections to serious invasive infections, such as pneumonia, endocarditis, bacteremia and sepsis [4,5]. It is estimated that Multi-drug resistant *Staphylococcus aureus* infections leads to high mortality with an associated annual health care costs [6,7]. Despite this high mortality rate, there are relatively few new antibacterial agents in the pharmaceutical pipeline [8]. Instead, the majority of antibiotics developed in the last decade are molecules re-engineered from existing antibiotic classes for which underlying resistance mechanisms are already present [9]. Therefore, effective new therapeutic options for treatment of infections caused by multidrug resistant *S. aureus* are urgently needed.

To the best knowledge of the authors, the literature is poor regarding nano titanium dioxide/gelatin effect on infected wounds, therefore, the aim of the present study was to evaluate effects of nano titanium dioxide /gelatin nanocomposite on infected wound healing in rats as an animal model study. The assessments were based on planimetric, histologic and histomorphometic features of the infected wounds.

## Materials and Methods

*Preparation and characterization of titatnium dioxide nanoparticles/gelatin composite*

**Fig. 1:**
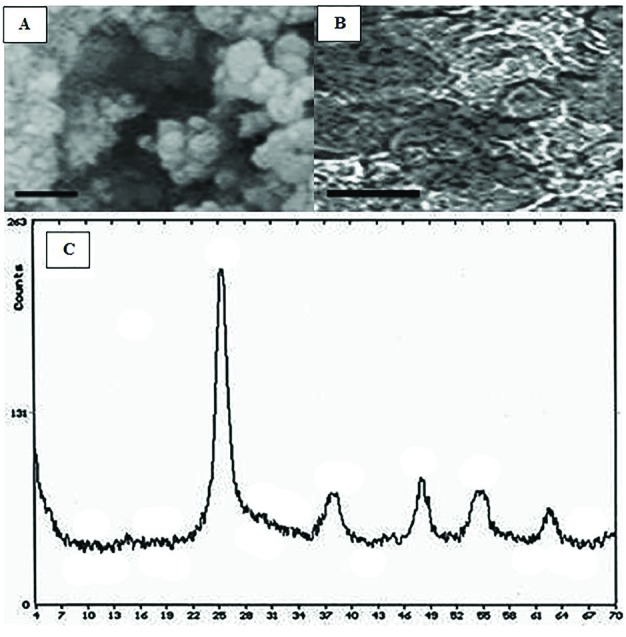
TEM image of titatnium dioxide nanoparticles (A), FESEM image of titatnium dioxide nanoparticles/gelatin composite (B), XRD pattern of titatnium dioxide nanoparticles (C). The morphology and size distribution of the prepared titatnium dioxide nanoparticles were observed by FESEM and TEM (B and C). The shape of the particles was observed almost sphere like morphology with 30-35 nm in size

For preparation of titanium dioxide, 0.5 g Titanium was reflected in 5 mL cloridric acid 37% at 80 °C for 3 days. Then, 2 g ethylenglycol was added to the solution and the remained acid was discarded and titanium trichloror was remained in the ethylene glycol. Ti(C_12_H_29_COO)_3_ was synthesized via addition of 4 gr abitic acid and gradual thermal elevation to 200 °C. Afterward produced gel was calcinated in 400 ºC. Nanomaterial morphology, size distribution and size were characterized by TEM (transmission electron microscope), FESEM (field emission scanning electron microscopy) and XRD (X-ray diffraction) (Figure 1). Nanotitanium dioxide anatase with the size of 30-35 nanometers was prepared. For gelatin production, 2 g gelatin was dissolved in 100 mL of distilled water and the solution was placed in cold water bath. Then, 0.5 g titanium dioxide was scattered in 100 mL of warm gelatin solution by ultrasound technique. Finally, with rapid mixing and putting in cold water bath, gel-based nanocomposite was prepared.

**Fig. 2:**
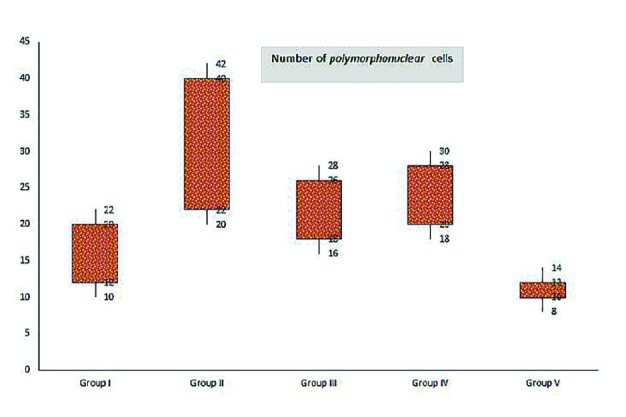
Box-and-whisker plots of number of polymorphnuclear cells in excisional model of the animals in experimental groups. Results were expressed as mean ± SEM

**Fig. 3:**
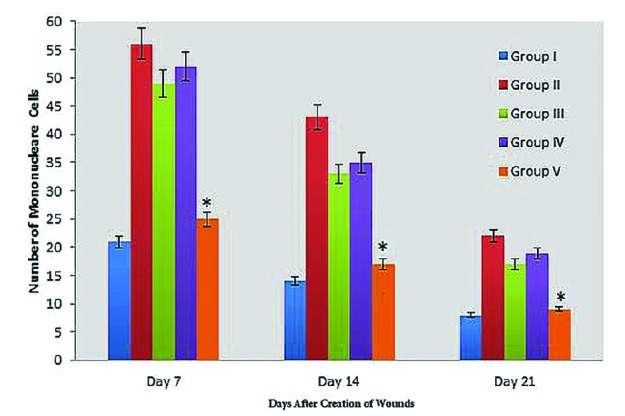
Bar graph indicating number of mononuclear cells in excisional model of the animals in experimental groups. Results were expressed as mean ± SEM. * *P* < 0.05 vs groups III and IV

**Fig. 4:**
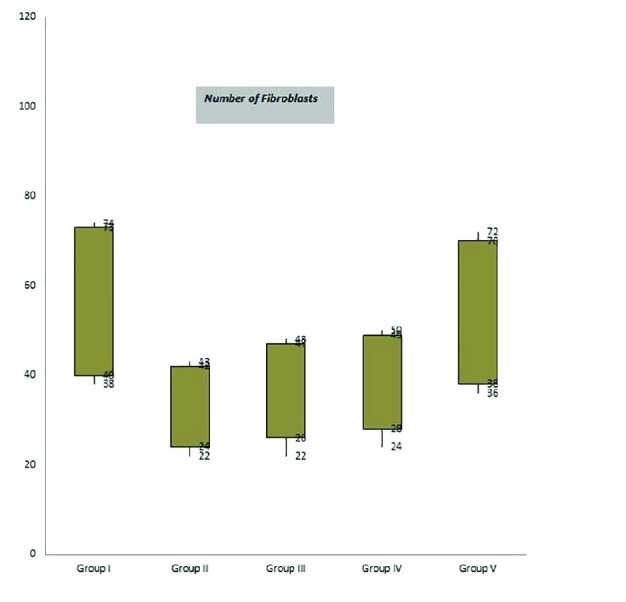
Box-and-whisker plots of number of fibroblasts in excisional model of animals in experimental groups. Results were expressed as mean ± SEM

**Fig. 5:**
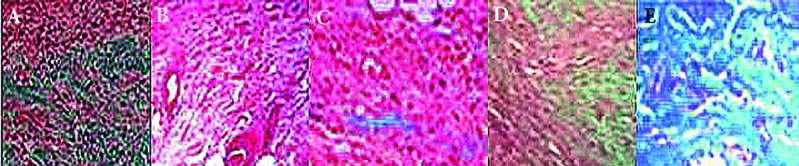
Representing micrographs of histological features of animal’s skin on day 14 after wound creation in excisional wound model. A: Group I, B: Group II, C: Group III, D: Group IV, E: Group V. Wounds with surrounding skin were prepared for histological microscopic evaluation by Masson trichrome staining (×400)

**Table 1:** Wound bacterial count in experimental groups on tow time points of day 7 and day 14

**Groups**	**Wound bacterial count (CFU** ^a^ **/g)**	
	**Day 7**	**Day 14**
**Group I**	0.00 ± 0.00	0.00 ± 0.00
**Group II**	1255.38 ± 278.10	1081.11 ± 274.28
**Group III**	1001.50 ± 215.20	970.37 ± 270.55
**Group IV**	1219.50 ± 230.80	980.60 ± 227.30
**Group V**	188.81 ± 45.29^b^	00.00 ± 0.00*

^a^CFU: Colony-forming units; ^b^*P*<0.05 *vs*. groups III and IV.

**Table 2:** Effects on circular excision wound contraction area (mm^2^). Values are given as mean ± SEM

**Wound area on days (mm** ^2^ **)**						
**Groups**	**6**	**9**	**12**	**15**	**18**	**21**
**Group I**	230.10±4.90	101.33±5.10	83.75±3.63	40.78±3.30	25.20±2.13	7.83±3.34
**Group II**	245.10±4.60	211.11±4.10	181.17±3.23	147.70±3.93	97.60±3.41	74.10±3.75
**Group III**	222.25±4.12	190.73±4.77	170.89±3.24	126.50±2.10	70.65±2.18	60.38±2.85
**Group IV**	221.21±4.13	191.55±4.50	175.30±3.13	126.50±3.45	73.10±2.10	66.60±2.23
**Group V**	110.10±3.17^ a^	70.67±2.10^ a^	31.75±2.30^ a^	13.37±1.58^ a^	4.55±0.75^ a^	0.00±0.00^ a^

The treated groups are compared by Student t test with other groups. ^a^: The mean difference was significant at the .05 level *vs*. groups III and IV.

**Table 3:** Evaluation of Intensity of histological parameters in experimental groups

** Groups** **Parameters**	**Days**	**Group I**	**Group II**	**Group III**	**Group IV**	**Group V**
**Epithelialization**	7	**-**	**-**	**-**	**+**	++*
	14	**+**	**+**	**+**	**++**	+++*
	21	**++**	**+**	**+**	**++**	++++*
**collagen production score**	7	**+**	**-**	**-**	**+**	++*
	14	**++**	**+**	**+**	**++**	+++*
	21	**++**	**+**	**+**	**++**	++++*
**Vascularization**	7	**+**	**-**	**-**	**-**	+++*
	14	**++**	**+**	**+**	**+**	++++*
	21	**++**	**+**	**+**	**+**	++++*
**Congestion**	7	**+++**	**+++**	**+++**	**+++**	+*
	14	**+**	**+++**	**++**	**+**	**-**
	21	**-**	**++**	**++**	**+**	**-**
**Acute Hemorrhage**	7	**+++**	**++++**	**+++**	**++** **+**	+*
	14	**++**	**+++**	**+++**	**+** **+**	**-**
	21	**-**	**++**	**++**	**+**	**-**

Classification of histological parameters according to the intensity of occurrence: - absence; + discrete; ++ moderate; +++ intense; ++++ very intense. Histopatological damages were assessed as explained under material and methods on days, 7, 14 and 21 of lesion. *P<0.05 *vs*. groups III and IV.

*Wound creation and infection*

Rats were anesthetized by an intraperitoneal injection of ketamine (70 mg/kg of BW) and xylazine (5mg/kg of BW), the hair on their back was shaved and the skin cleansed with 70% alcohol solution. Following shaving and aseptic preparation, a circular excision wound was made by cutting away approximately 115 mm^2^ full thickness of predetermined area on the anterior-dorsal side of each rat. Small gauze was placed over each wound and then inoculated with 5 × 10^7^ CFU of *Staphylococcus aureus *ATCC 43300. The methicillin-resistant *S. aureus *ATCC 43300 strain was commercially available. The rats were returned to individual cages and they were examined daily. After 24 h, the wounds were opened, the gauze removed and treatment started. 

*Antibacterial testing*

The antibacterial activity of titanium dioxide /gelatin nanocomposite and gelatin were tested qualitatively. *Staphylococcus aureus* were used for testing the antimicrobial activity of the samples. They were cultured on nutrient agar and incubated at 37 °C for 48 h. The antibacterial activity was tested using modified agar diffusion assay (Well diffusion method). The plates were examined for possible inhibition zone after 48 h incubation. The presence of any clear zone around samples on the plates was recorded as an inhibition against the microbial species. The antibacterial activities of each sample were repeated three times.

*Study design and animals*

The present study was registered under ethical code 91000431 in AJA University of Medical Sciences Committee of Ethic.. This study was carried out in strict accordance with the guidelines of the Ethics Committee of the International Association for the Study of Pain [11]. All bacterial inoculations and treatments were performed under conditions to minimize any potential suffering of the animals. Fifty male rats were randomized into five groups of ten animals each. In group I, 0.1 mL sterile saline 0.9% solution was added to the wounds with no infection. In group II, the wounds were infected with MRSA and only treated with 0.1 mL the sterile saline 0.9% solution. In group III, infected wounds were treated with gelatin. In group IV, animals with infected wounds were treated with 0.1 mL titanium dioxide nanoparticles. In group V, animals with infected wounds were treated with titanium dioxide /gelatin nanocomposite. All the test formulations were applied for 7 days, starting from the first treatment. 

*Microbiological assessments*

Briefly, for total bacterial count on days 7 and 14 of treatment after wound creation the granulated tissues were excised aseptically. Then, 0.1 g of sample was crushed and homogenized in sterile mortar containing 10 ml of sterile saline. The homogenized sample was serially diluted in tube containing 9 ml of sterile saline to 10-5. The diluted samples were cultured on plate count agar (Merck KGaA, Darmstadt, Germany) superficially and duplicated. The cultured plates were incubated at 37 ºC for 24 to 48 hours. After incubation, all colonies were counted and results described as CFU/g of granulation tissue [12].

*Excision wound model and wound area measurements*

Wound-healing property was evaluated by wound contraction percentage and wound closure time. Photographs were taken immediately after wounding and on days 6, 9, 12, 15, 18 and 21 post-wounding by a digital camera while a ruler was placed near the wounds. The wound areas were analyzed by Measuring Tool of Adobe Acrobat 9 Pro Extended software (Adobe Systems Inc, San Jose, CA, USA) and wound contraction percentage was calculated using the following formula: Percentage of wound contraction = (A_0_ – A_t_) / A_0_ × 100 

Where A_0_ is the original wound area and A_t_ is the wound area at the time of imaging. Animal houses were in standard environmental conditions of temperature (22 ±3°C), humidity (60 ± 5%), and a 12h light/dark cycle. The animals were maintained on standard pellet diet and tap water. All rats were closely observed for any infection and if they showed signs of infection were separated, excluded from the study and replaced**.**

*Histology and morphometric studies*

The tissue samples were taken on 7, 14, 21 days after surgery from periphery of the wound along with normal skin and fixed in 10% buffered formalin, drhydrated and embedded in paraffin wax, sectioned at 5 µm and stained with hematoxylin and eosin (H&E) and Masson’s trichrome stains. Photomicrographs were obtained under light microscope to assess the predominant stage of wound healing. Three parallel sections were obtained from each specimen. Cellular infiltration including the number of mononuclear cells, poly morphonuclear cells and fibroblastic aggregation were quantitatively evaluated. Acute hemorrhage, congestion, vascularization, epithelialization, collagen production and density were also evaluated qualitatively. Morphological findings were scored using image analyzing software (Image-Pro Express, version 6.0.0.319, Media Cybernetics, Silver Springs, MD, USA). The histological parameters were classified according to the intensity of occurrence in five levels (- absence; + discrete; ++ moderate; +++ intense; ++++ very intense) [13].

*Determination of hydroxyproline levels*

On the day 21 after surgery, a piece of skin from the healed wound area was collected and analyzed for hydroxyproline content. As a major part of collagen, hydroxyproline has an essential role in collagen stability. The collagen is the major component of extracellular tissue, which gives support and strength. Tissues were dried in a hot air oven at 60–70 ◦C to constant weight and were hydrolyzed in 6N HCl at 130 ◦C for 4 h in sealed tubes. The hydrolysate was neutralized to pH 7.0 and was subjected to Chloramine-T oxidation for 20 min. The reaction was terminated by addition of 0.4M perchloric acid and color was developed with the help of Ehrlich reagent at 60 ºC and measured at 557 nm using UV-visible spectrophotometer.

*Statistical analysis*

Differences among groups were evaluated by Kruskal–Wallis variance analysis. When the P-value from the Kruskal–Wallis test statistics was statistically significant, multiple comparison tests were used to know differences. Comparison among days was assessed by Mann–Whitney U-test. The Bonferroni correction was applied for all possible multiple comparisons. SPSS 18 (SPSS Inc., Chicago, IL, USA) was used for statistical analysis. A *P*-value was set at 0·05.

## Results

*Microbiological assessments*

In animals of group V whose infected wounds were treated with titatnium dioxide nanoparticles/gelatin composite, the counts of *S. aureus* cultured in the wound tissues were significantly lower than in the infected wounds in groups III and IV (*p*=0.001).

No animals died due to infection or anesthetics. The uninfected wounds treated with saline had no CFU/g of *S. aureus* count. Topical application of titanium dioxide /gelatin nanocomposite significantly reduced the rate of total bacterial count on 7 and 14 days post-wounding compared to groups III and IV (*p*=0.001) (Table 1). 

*Reduction in wound area*

Wound contraction percentage in different groups within the study period is shown in Table 2. The healing rate of wounds in group V was significantly different compared to groups III and IV (*p*=0.001).

*Histological and morphometric findings*

There were significant differences in comparisons of group V and IV, particularly in terms of cellular infiltration, acute hemorrhage, congestion, edema, collagen production and density, reepithelialisation and neovascularization. During the study period, scores for reepithelialisation and neovascularisation were significantly higher in group V rats than groups III and IV (*p*=0.001). Polymorphonuclear (PMN) and mononuclear cell (MNC) count, fibroblast cell proliferation and also Mean Rank of the qualitative study of acute hemorrhage, vascularization, conjestion, edema and collagen production score in group V were significantly higher than those of groups III and IV (*p*=0.001) (Table 3) (Figures 2, 3,4 and 5).

*Hydroxyproline content of the wound*

Proline is hydroxylated to form hydroxyproline after protein synthesis. Hydroxyproline contents in groups I to V were found to be 44.78±3.54, 61.54±2.25, 70.62±3.62, 73.11±2.23 and 97.88±3.77 mg g^-1^, respectively. Hydroxyproline contents were significantly increased in the group V which implies more collagen deposition compared to groups III and IV (*p*=0.001). 

## Discussion

Inflammation, proliferation and tissue remodeling are three phases of healing process which occur following tissue damages as closely as possible to its natural state. The healing process is activated when platelets come into contact with exposed collagen leading to platelet aggregation and the release of clotting factors resulting in the deposition of a fibrin clot at the site of injury. The fibrin clot serves as a provisional matrix and sets the stage for the subsequent events of healing. Inflammatory cells also arrive along with the platelets at the injury site providing key signals known as growth factors. The fibroblast is the connective tissue cell responsible for collagen deposition required to repair the tissue injury. The collagen is the main constituent of extra cellular tissue, which is responsible for support and strength [14].

Nanoparticles have become significant in the regenerative medicine field in the last two decades [15]. Many biological processes happen at through mechanisms that fundamentally act at the nanometer scale. Thus, materials such as nanoparticles can be used as unique tools for drug delivery, imaging, sensing, and probing biological processes [16]. In the context of wound healing, the special properties of nanoparticles like electric conductivity, antimicrobial activity, and high surface to volume ratio, swelling, and contraction make nanoparticles versatile resources.

NanoTio2 has reported to govern progression of healing process through enhancement of skin moisture (hydrophilic activity) and antimicrobial properties [17]. In the present study the healing effects of titatnium dioxide nanoparticles/gelatin composite on infected wound was studied. The findings of the present study showed that titatnium dioxide nanoparticles/gelatin composite treated wounds were significantly contracted in second and third weeks of the experiment compared to other groups.

The highest levels of wound contraction in the third week was observed in titatnium dioxide nanoparticles/gelatin composite treated group compared to other groups that represented promising effect of titatnium dioxide nanoparticles/gelatin composite on wound contraction and closure. High and significant contraction levels in gelatin group in comparison with control might be associated with hydrophilic effect of gelatin and preservation of wound moisture that improved wound healing [17].

Fibroblast numbers is a well-known index for quality assessment of connective tissue healing. Presence and early proliferation of fibroblasts in Tio2 group demonestrated growth stimulation of fibroblasts by Tio2. Others conducted a survey on the effects of nanocomposite of chitosan-titanium dioxide on excisional wound and suggested healing improvement by cell growth stimulation of nanoTio2 [18,19]. Fibroblast creates extracellular matrix and collagen that play essential role in wound healing phases [20].

Inflammatory phase is the first step of wound repair and is determined by the presence of inflammatory cells. This phase is critical due to association of proliferative, reepithelization, contraction and wound closure with cytokines secreted in inflammatory phase [21]. In the present study group V showed the least numbers of inflammatory cells on day 7 that could be associated with anti-inflammatory and antibacterial activity of NanoTio2 that could reduce polymorphonuclear (PMN) cells via reduction in secondary infection and acceleration in inflammation initiation and termination [17]. Presence of few numbers of PMN cells and high numbers of fibroblast on day 7 in group V indicated healing acceleration in titatnium dioxide nanoparticles/gelatin composite treated animals.

Reepithelialisation makes a barrier between wound and environment in wound healing [22]. New formed epithelium is characterized by more cells and layers compared to normal epithelium. As soon the wound surface is covered by new epidermal cells, differentiation initiates, cell shape changes to normal and rearrangement and reduction of cell layer occurs [23]. Based on the findings of the present study, epithelialization in group V was approximately complete (96/6%) on day 14 and one week earlier than others followed by complete epithelialization (100%) on the last day of the experiment. This outcome was indicative of epithelialization stimulation effect of titatnium dioxide nanoparticles/gelatin composite that was consistent with those of other studies [18]. 

Despite sterility of wounds upon creation, due to loss of epithelial integrity, the wound is disposed to infection with failure in healing and mortality in more severe cases as wound infection consequences. Presence of neutrophils in histopathological sections is indicator of wound infection [24].

According to the findings of the present study group V on days 7 and 14 showed the least infection, existed only on scab and epidermis and no infection was observed on day 21. It could be concluded that titatnium dioxide nanoparticles/gelatin composite had antibacterial properties that was in agreement with those of other studies [25,26]. In the present study, antibacterial effect of titatnium dioxide nanoparticles/gelatin composite were detected more than other groups. Early epithelialization in group V prevented wound from infection and penetration of microorganisms to the healing tissue. Both titanium dioxide nano particles and gelatin as the carrier exhibited hydrophilic feature and accelerated healing process. 

Enhancement of wounds is associated with reepithelialisation, fibroblast proliferation and angiogenesis [27]. Since the pathological phases of wound healing process have dynamic feature and results of inflammatory, proliferative and remodeling phases are dependent on each other, for appropriate conclusion, all the healing factors should be considered together. Based on the results of the present study, high percentage of wound contraction and total pathological features as well as insignificant or absence of infection was observed in group V that was in agreement with those of other studies [20,28].

In conclusion, based on the results of the present study it could be concluded that titatnium dioxide nanoparticles/gelatin composite offered potential advantages in wound healing acceleration and improvement through fibroblast proliferation, and acceleration in wound repair associated with earlier wound contraction and stability of damaged area by rearrangement of granulation tissue and collagen fibers. In addition, antibacterial activity of Tio2 nanoparticles prevented wound infection and hydrophilic effect of gelatin and preservation of wound moisture improved wound healing. Therefore, titatnium dioxide nanoparticles/gelatin composite could be considered as a novel therapeutic option in wounds.
